# Evaluation of a Pseudotyped Virus Neutralisation Test for the Measurement of Equine Influenza Virus-Neutralising Antibody Responses Induced by Vaccination and Infection

**DOI:** 10.3390/vaccines8030466

**Published:** 2020-08-21

**Authors:** Rebecca Kinsley, Stéphane Pronost, Manuelle De Bock, Nigel Temperton, Janet M. Daly, Romain Paillot, Simon Scott

**Affiliations:** 1 Viral Pseudotype Unit (VPU), Medway School of Pharmacy, Universities of Kent & Greenwich, Chatham Maritime ME4 4TB, UK; rk571@cam.ac.uk (R.K.); n.temperton@kent.ac.uk (N.T.); 2 LABÉO Frank Duncombe, 1 route de Rosel, 14053 Caen CEDEX 4, France; stephane.pronost@laboratoire-labeo.fr; 3 Normandie University, UNICAEN, BIOTARGEN EA7450, 14280 Saint-Contest, France; 4 Elanco Animal Health, Plantin en Moretuslei, B-2018 Antwerpen, Belgium; de_bock_manuelle@elanco.com; 5 School of Veterinary Medicine and Science, University of Nottingham, Sutton Bonington LE12 5RD, UK; janet.daly@nottingham.ac.uk; 6 Animal Health Trust, Centre for Preventive Medicine, Lanwades Park, Kentford Newmarket CB8 7UU, UK

**Keywords:** equine influenza, horse, pseudotype virus, vaccine

## Abstract

Equine influenza is a major respiratory disease of horses that is largely controlled by vaccination in some equine populations. Virus-neutralising antibodies, the mainstay of the protective immune response, are problematic in assaying for equine influenza virus, as most strains do not replicate efficiently in cell culture. Surrogate measures of protective antibody responses include the haemagglutination inhibition (HI) test and single radial haemolysis (SRH) assay. For this study, a pseudotyped virus, bearing an envelope containing the haemagglutinin (HA) from the Florida clade 2 equine influenza virus strain A/equine/Richmond/1/07 (H3N8), was generated to measure HA-specific neutralising antibodies in serum samples (*n* = 134) from vaccinated or experimentally-infected ponies using a pseudotyped virus neutralization test (PVNT). Overall, the results of PVNT were in good agreement with results from the SRH assay (100% sensitivity, 68.53% specificity) and HI test (99.2% sensitivity, 49.03% specificity). The PVNT was apparently more sensitive than either the SRH assay or the HI test, which could be advantageous for studying the antibody kinetics, particularly when antibody levels are low. Nevertheless, further studies are required to determine whether a protective antibody level can be defined for the SRH assay and to ascertain the inter-laboratory reproducibility. In conclusion, the PVNT efficiently measures neutralising antibodies after immunization and/or experimental infection in the natural host, and may complement existing antibody assays.

## 1. Introduction

Equine influenza (EI) is a major respiratory disease of horses (*Equus ferus caballus*) caused by influenza A virus (IAV), which can result in important welfare issues and significant loss for the equine industry, as illustrated in Australia in 2007 [1] and more recently in the United Kingdom, when horse racing was stopped for six days in early 2019. Vaccination is used globally to prevent equine influenza virus (EIV) infections [1,2]. Since the first inactivated whole virus vaccines were introduced in the 1960s, various vaccine types have been developed, with inactivated whole virus, subunit, live-attenuated, and viral-vector-based vaccines in current use (reviewed by Paillot, 2014 [1]). Protection elicited by IAV vaccination requires stimulation of a humoral immune response and cell-mediated immunity required for virus-infected cell clearance. A neutralising antibody (NAb) against the major viral surface protein, haemagglutinin (HA), is one of the primary vaccine-induced lines of defence, because it prevents IAV cell binding and entry [3]. In the human field, the contribution to protection of NAb against the other surface protein, neuraminidase (NA), which interferes with the release of virions [3], has been increasingly recognized [4]. As for human seasonal influenza vaccines, the strain content of EIV vaccines is reviewed annually and updated periodically to maintain vaccine effectiveness. In the last decade, circulating EIV has belonged to one of two clades in the Florida lineage of the H3N8 subtype (FC1 or FC2). The latest recommendation from the World Organisation for Animal Health (OIE) Expert Surveillance Panel on EI Vaccine Composition is that equine influenza vaccines should contain both a clade 1 virus (represented by A/equine/South Africa/04/2003-like or A/equine/Ohio/2003-like viruses) and a clade 2 virus (represented by A/equine/Richmond/1/2007-like viruses) (https://www.oie.int/scientific-expertise/specific-information-and-recommendations/equine-influenza/).

Virus-neutralization tests are widely regarded as the gold standard for measuring protective antibody responses to virus vaccination. However, influenza A viruses typically do not induce visible cytopathic effects (CPE), which is the usual read-out for cell culture-based virus neutralization tests. Furthermore, most EIV isolates replicate poorly in cell cultures, and the alternative approach of performing neutralization tests in embryonated hens’ eggs is extremely cumbersome. For these reasons, the haemagglutination inhibition (HI) test, which measures inhibition of HA binding to receptors on red blood cells, has been the mainstay of testing antibody responses to influenza A viruses [5]. The single radial haemolysis (SRH) assay, which measures complement-mediated lysis of red blood cells coated with whole virus, was introduced as a more reproducible assay for measuring influenza antibodies. Measurement of SRH antibody levels provides a well-established correlate of protection against EIV infection, with defined thresholds associated with virus shedding reduction and clinical protection [6,7,8]. More recently, pseudotyped viruses (PVs) based on lentivirus vectors, have been generated to measure equine influenza neutralising antibodies in horse serum and have demonstrated reasonable correlation (correlation coefficient *r* = 0.65) with the SRH assay for detecting antibody levels [9,10]. PVs are non-replicating, hybrid viruses with the disabled core of a vector virus and the envelope protein (or proteins) of the study virus (e.g., influenza virus haemagglutinin). The PVs also incorporate a reporter gene, such as firefly luciferase, flanked by lentivirus long terminal repeats. This leads to the reporter gene being inserted into the genome of transduced target cells and subsequently expressed, providing a direct quantitative readout of the amount of virus entering the cells, which is a major advantage for viruses like EIV that do not cause CPE.

The current study aimed to evaluate a pseudotyped virus neutralization test (PVNT) for measurement of EIV-specific NAb response in the natural host following vaccination or experimental infection with EIV, and to compare with responses measured by SRH or HI assays.

## 2. Materials and Methods

### 2.1. Serum Samples

Two sets of serum samples obtained from vaccination and experimental infection studies unrelated to this work were used in this study. The first set contained 108 samples collected from 27 Welsh mountain ponies (*Equus ferus caballus*, Welsh section A breed, also called Welsh Mountain Pony breed, United Kingdom) vaccinated with two doses of vaccine and sampled at four time points (Table 1). The sampling time points were day 0 (D0: when the first vaccine dose was given), day 28 (D28: when a second vaccine dose, V2, was given), day 56 (D56: 4 weeks after V2), and day 238 (D238: 30 weeks after V2). Groups 1–4 received two doses of non-commercial, inactivated whole virus vaccine containing either A/equine/Richmond/1/2007 (FC2) or A/equine/Kentucky/2009 (FC1). Group 5 received phosphate-buffered saline (PBS) only, and group 6 were vaccinated with ProteqFlu, a commercial canarypox-vectored vaccine expressing only the HA protein of equine influenza virus strains A/equine/Ohio/2003 (FC1) and A/equine/Richmond/1/2007 (FC2).

The second serum set contained 26 serum samples from Welsh mountain ponies with mixed EI vaccination history, sampled 14 days after experimental infection with influenza A/equine/Cambremer/2012 (H3N8 FC2). Ponies had recovered from the infection at the time of sampling. Both sets of sera were stored at −20°C prior to use. 

### 2.2. Single Radial Haemolysis (SRH) Assay

**Figure 1:**
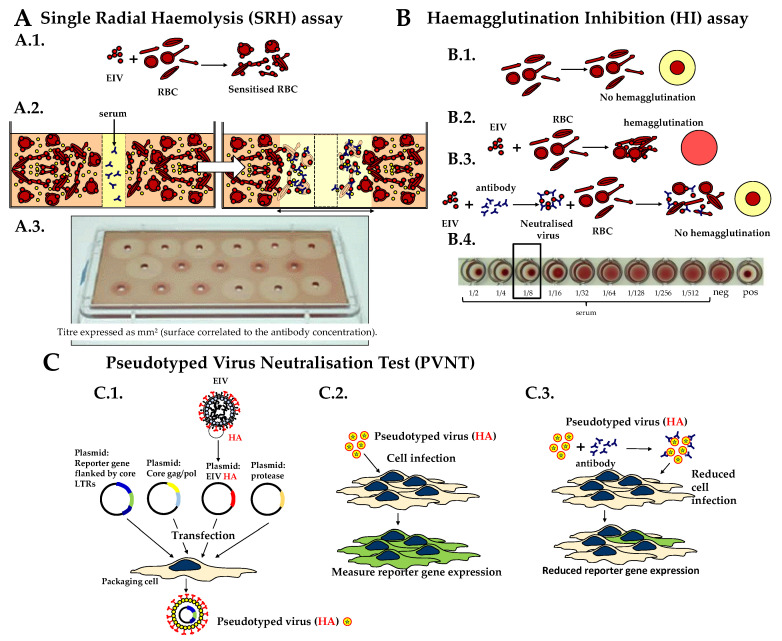
Serological assay/tests to measure equine influenza virus (EIV)-specific antibody response. (**A**) Single radial haemolysis (SRH) assay: (A.1.) sheep red blood cells (RBCs) are sensitized with EIV, (A.2.) EIV-sensitized RBCs are mixed with guinea-pig complement and melted agar, and (A.3) poured onto an immunoplate. When the gel is set, 10 µL of heat-inactivated undiluted serum is placed in the punched hole. The plate is incubated for 20 to 24 h at 30 °C to allow serum diffusion prior to measurement of the haemolysis area. (**B**) Haemagglutination inhibition (HI) test: (B.1.) in the absence of EIV, chicken RBC sediment to the bottom of round-bottomed wells of a 96-well plate; (B.2.) in the presence of EIV and RBC agglutinate; (B.3.) the incubation of EIV with a serum containing EIV-specific antibody prevents the RBC agglutination; (B.4) an example of a serum titration (i.e., HI titer of 1:8). (**C**) Pseudotyped virus neutralisation test (PVNT), adapted from ref [9]: (C.1.) to generate lentivirus pseudotypes, four plasmids expressing EIV HA, HIV gag-pol, firefly luciferase reporter gene, and an HA-cleaving protease (e.g., TMPRSS2) are transfected into a packaging cell. EIV HA pseudotyped particles are released in the supernatant, harvested, then titrated on an influenza-permissive cell line. (C.2.) The level of reporter gene expression is measured in this target cell culture. (C.3.) The pre-incubation of influenza HA-pseudotyped virus with a serum containing EIV-specific antibody reduces cell infection and the associated reporter gene expression.

Antibodies were measured by single radial haemolysis (SRH) assay [5] against the EIV strain A/equine/Richmond/1/2007 (H3N8), as previously described [5] (Figure 1A). Control antiserum against A/equine/South Africa/4/2003 (H3N8; Florida Clade 1; ref EU SA/4/03 Y0000712) from the European Directorate for the Quality of Medicines and Healthcare (EDQM) was included on each plate [11]. The amount of SRH antibody is expressed as the area of haemolysis (mm²).

### 2.3. Haemagglutination Inhibition (HI) Test

HI antibody titers were expressed as the last dilution of serum that inhibited chicken red blood cell haemagglutination by A/equine/Richmond/1/2007 (H3N8), as previously described [5] (Figure 1B). As mentioned in the OIE Terrestrial Manual, a cut-off point for positive samples has not been determined for the HI test [5]. In the current study, a titer >1:8 was regarded as positive. For statistical and graphical reasons, an HI titer <1:8 was converted to 4. Values for in-house reference serum standards were within the expected range.

### 2.4. Pseudotyped Virus Neutralization Test (PVNT)

Lentivirus particles pseudotyped with the A/equine/Richmond/1/2007 HA were produced as described in Scott et al., 2016 [10]. Briefly, four plasmids expressing the EIV HA gene, an HA-cleaving protease (i.e., TMPRSS2), the HIV gag-pol genes, and a manipulated HIV genome containing the luc reporter gene replacing endogenous genes, were co-transfected into HEK293/17 (PV producer) cells (see Figure 1C). Exogenous neuraminidase was added after 24 h to allow viral particle release, with supernatant harvesting after a further 24 h. PVNTs were conducted as previously described [9] (Figure 1C). Briefly, PV diluted to produce a signal of 10^6^ relative light units (RLUs) was incubated with serially two-fold diluted serum samples for 1 h at 37 °C before HEK293T/17 cells were added and plates incubated for 48 h. No serum and cell only controls were also included. Luciferase expression was quantified using BrightGlo reagent (Promega), and the serum titres obtained expressed as a 50% inhibitory concentration (logIC50).

### 2.5. Statistical Analysis

All statistical analysis was performed using StatGraphics Centurion XVI Version 16.1.12 for windows (StatPoint Technologies, Inc). Significance levels were set at *p* < 0.05. The receiver operating characteristic (ROC) curves were generated and analyzed using the web-based calculator for ROC curves (Eng J. ROC analysis: web-based calculator for ROC curves; Baltimore: Johns Hopkins University (updated 19 March 2014), available from http://www.jrocfit.org).

## 3. Results

### 3.1. Defining PVNT Threshold for Positivity

**Figure 2:**
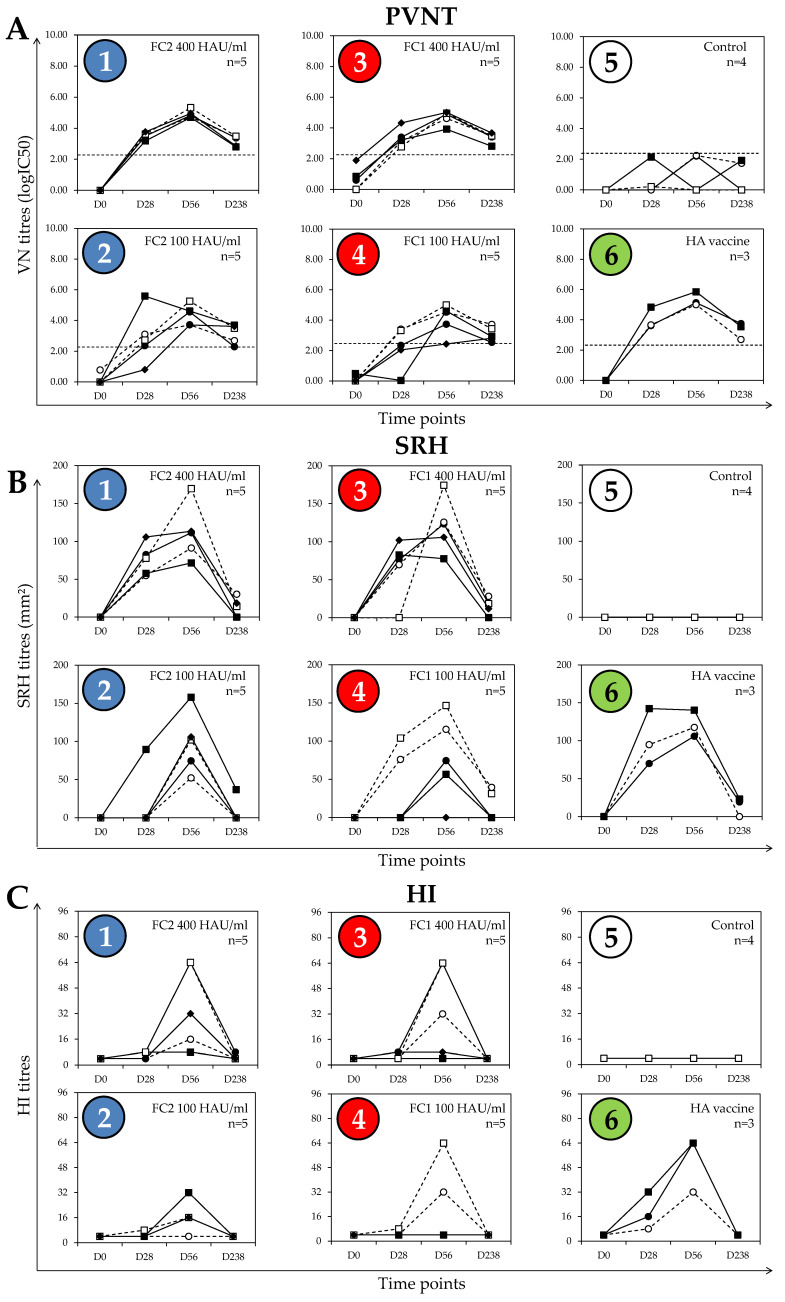
Serum set #1 antibody response kinetics per group, measured by (**A**) pseudotype virus neutralization test (PVNT), (**B**) single radial haemolysis (SRH), and (**C**) hemaglutination inhibition (HI) assays. The dotted line represents the calculated positive threshold (2.21 logIC50) for the PVNT. The number of ponies per group is indicated (*n*). D values indicate days after immunization. HAU/mL = HA unit per mL. Groups 1 and 2 are indicated in blue, groups 3 and 4 are indicated in red, and the control group 5 and HA vaccine group 6 are indicated in white and green, respectively.

All 27 D0 samples in serum set #1 tested negative for EIV antibodies in the SRH assay (<3 mm^2^) and HI test (<1:8). The mean logIC50 for these samples was 0.2 ± 0.4, with all samples below 1.9 logIC50, which was previously considered as the positivity threshold [9]. As expected, unvaccinated control ponies (group 5) remained seronegative by SRH assay and HI test at all time points (Figure 2). While the logIC50 remained low for these animals, the mean logIC50 was 0.7 ± 1.0 for samples from D0 to D238 (*n* = 16), and four serum samples reached 1.9 logIC50. The distribution of logIC50 results obtained for these seronegative serum samples when measured by SRH or HI (*n* = 39) indicates that the 95% upper limit in the PVNT is a neutralising antibody titer of 2.21 logIC50, which was therefore used as the threshold for positivity in this report.

### 3.2. Post-Vaccination Antibody Responses

Sera from all ponies in groups 1 and 3 of serum set #1, which received the higher dose of the monovalent vaccines, had antibody levels above the threshold in the PVNT at 4 weeks after the first dose of vaccine (D28). All horses in group 1, and four of the five horses in group 3, were positive in the SRH assay at D28, whereas only two ponies in group 1 and one pony in group 3 had positive HI titers (1:8) at D28 (Figure 2).

In all of the groups of ponies that received a vaccine, the peak titer measured in the PVNT occurred on D56 (4 weeks after the second vaccine dose), except for one pony in group 2, which received the low dose of the FC2 vaccine and had the highest logIC50 on D28 (indicating a good response to priming immunization). The PVNT response at D56 was lower for this specific pony, which tends to indicate that the peak occurred earlier after V2 and that the response had started to decline when measured at this time point. This pony was the only member of group 2 to have a measurable SRH antibody response on D28, with high SRH and HI antibody titers at D56 when compared with the ponies from the same group, which also indicates a good response to both priming and boost immunization (V1 and V2, respectively). The peak HI antibody titres all occurred at D56.

Although the PVNT titres declined between D56 and D238 in the majority of vaccinated ponies, most remained above the positive threshold value of 2.21 logIC50. The SRH values also declined between D56 and D238, with only nine ponies remaining seropositive at D238 (three, four, and two ponies in groups 1, 3, and 6, respectively). In terms of the HI antibody response, five ponies in groups 1 to 4 remained seronegative at all time points, and only one pony (in group 1) had a measurable HI titre of 1:8 on D238.

Multifactorial analyses indicated that the vaccine dose had a significant effect on the logIC50 measured by PVNT from D28 to D238 (D0 excluded, *p* = 0.0106 with time point and EIV strain effects removed). The EIV vaccine strain had no significant effect on logIC50 when measured from D28 to 238 (D0 excluded, *p* = 0.300 with time point and dose effects removed). Multifactorial analysis of SRH antibody values gave similar results to those obtained for the PVNT antibody response; the vaccine dose had a significant effect on the SRH value when measured from D28 to D238 (D0 excluded, *p* = 0.0063 with time point and EIV strain effects removed). As for the PVNT antibody response, the EIV strain had no significant effect on the SRH antibody value when measured from D28 to 238 (D0 excluded, *p* = 0.88 with time point and dose effects removed). These results indicate that both PVNT and SRH tests were responsive to the dose of vaccine administered, with no measurable effect of the EIV strain incorporated in the vaccine used. The low level of HI-positive serums at D28 and D238, combined with a large response heterogeneity at D56 (e.g., from 4 to 64 in groups 1, 3, and 4) did not allow any meaningful statistical analysis.

### 3.3. Comparison of PVNT with SRH and HI

For comparison of the results obtained by PVNT with those obtained by SRH and HI, results from both serum sets were used (134 serum samples). The 26 serum samples from serum set #2 (collected 2 weeks after experimental infection with EIV) were all positive by PVNT, with logIC50 values ranging from 3.94 to 11.61. They were also all positive by SRH (98.6 to 204.1 mm²) and HI (1:8 to 1:1028) (Table S1). These results confirmed the seroconvertion induced by the experimental infection with the EIV strain A/equine/Cambremer/2012.

**Figure 3:**
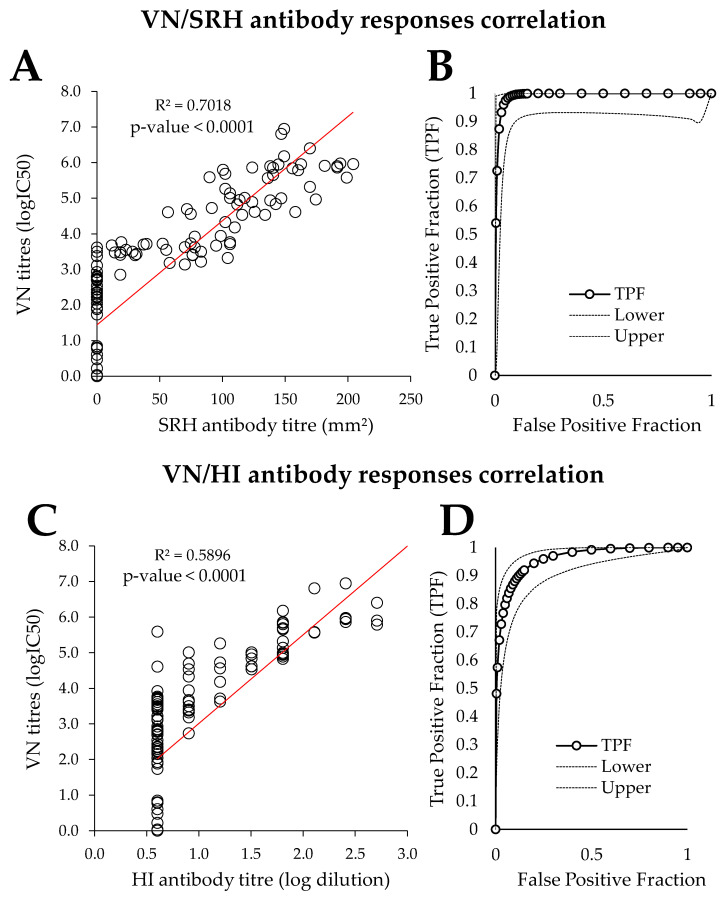
Pseudotype virus neutralization (VN)/single radial haemolysis (SRH) and VN/hemaglutination Inhibition (HI) assay ((**A**,**C**), respectively), as well as receiver operating characteristic (ROC) curve analysis ((**B**,**D**), respectively). Results from both serum sets were used (*n* = 134). TPF: true positive fraction.

The direct correlation analysis between the SRH and PVNT results provided a correlation coefficient *r* and a determination coefficient *R*² of 0.84 and of 0.70, respectively (*n* = 134) (Figure 3A). The direct correlation analysis between the HI and VN antibody titres provides a correlation coefficient *r* and *R*² values of 0.77 and 0.59, respectively (*n* = 134) (Figure 3C). As observed with the SRH comparison, PVNT performed well at time points where the HI antibody response was of low amplitude (D28 and D238 in all EI vaccine groups). At time points where HI antibody titres were measurable, PVNT results correlated well with the HI antibody titres.

Results from both sets of samples were also used to compare the PVNT with the SRH assay (considered as gold standard for the analysis) by ROC curve analysis (Figure 3B). The calculated area under the curve (AUC) was high at 0.9906 (99.06 ± 0.008%), which indicated a very good performance of the PVNT. According to this analysis, the positive threshold value of 2.21 logIC50, defined in Section 3.1, gave 100% sensitivity and 68.53% specificity (sensitivity is the proportion of true-positives that actually test positive, and how well a test is able to detect positive individuals in a population; specificity is the proportion of true-negatives that actually test negative, and reflects how well an assay performs in a group of disease negative individuals). The specificity value could be explained by the higher sensitivity of the PVNT, with 19 serum samples ≥2.21 logIC50 but negative by SRH assay. A PVNT threshold value of 3.14 logIC50 was associated with 97.47% sensitivity and 95.05% specificity. The ROC curve analysis for the PVNT against the HI test (considered as gold standard for this analysis) calculates an AUC of 0.9584 (95.84 ± 0.016%) that also confirms a good performance of the PVNT (Figure 3D). The positive threshold value of 2.21 logIC50 defined in Section 3.1 gave 99.2% sensitivity and 49.03% specificity, which could be explained by the higher sensitivity of the PVNT, with 43 serum samples ≥2.21 logIC50 but negative by HI test. A PVNT threshold value of 3.14 logIC50 is associated with 97.47% sensitivity and 95.05% specificity. Above a PVNT threshold of 3.4 logIC50, the sensitivity decreases to below 95%, while the specificity is 79.34%.

## 4. Discussion

Pseudotyped viruses presenting the surface proteins of numerous viruses have been exploited for different applications: from basic cell tropism and receptor studies [12,13], through to antiviral screening, serological surveillance [14,15] vaccine efficacy evaluation, and even as vaccine antigens themselves [16,17,18]. A major use of PVs is in the sensitive measurement of neutralising antibody responses in sera following natural infection or vaccination. In a previous pilot study, the PVNT demonstrated reasonable correlation (*r* = 0.65) with SRH for detecting antibody levels in equids [9]. In the current study, the PVNT was applied to a larger sample set, and antibody detection compared with both SRH and HI.

### 4.1. Defining PVNT Threshold for Positivity

In the pilot study from Scott et al. [10], a threshold of 1.9 logIC50 was considered as the positivity threshold for the PVNT, using a PV displaying the HA of A/equine/Sussex/1989 (H3N8) [9]. This was redefined in the current study using pre-vaccination serum samples and serum samples from unvaccinated ponies as 2.21 logIC50. Although this value was associated with 100% specificity (using SRH as the gold standard), specificity was only 68.53% on ROC analysis. Defining a threshold value of 3.14 logIC50 gave 95% specificity and 97.5% sensitivity. This suggests that the positive threshold value should be carefully evaluated before applying the PVNT to study antibody responses. Further studies are also required to determine whether clinical and/or virological antibody thresholds can be assigned to the PVNT (thresholds above which clinical signs of disease and virus shedding are significantly reduced, respectively).

### 4.2. Post-Vaccination Antibody Responses

As previously observed [9], using the PVNT provides measurable titers at time points when the SRH antibody response is of low amplitude (example of SRH antibody kinetics described in [19,20,21]), such as on D28 in both low-dose vaccine groups (groups 2 and 4) and at D238 in all vaccinated groups. The ability of PVNT to measure a neutralising antibody titre when both SRH assay and HI test reached their limit of detection will be useful to define antibody response and kinetics at the early stages of immunization (i.e., in the days and weeks after V1), as well as the decrease of maternal-derived antibodies (MDAs) acquired by foals through colostrum consumption (when the mare is EI-vaccinated in the last week of gestation). Such data may be useful to avoid EI vaccination in the presence of MDA, which is recommended for most EI vaccines and has been shown to decrease the immunogenicity of some [1,19,22,23,24].

The availability of serum samples collected seven months after a second dose of vaccine (D238 of the study) allowed the performance of the PVNT during the “immunity gap” to be tested. This is the well-characterized period of susceptibility to EI infection between the second and third immunizations of a standard vaccination protocol when EIV-specific SRH antibody titers decrease sharply below the clinical protection threshold [6,7]. At this time point, the PVNT appears to be more sensitive than the SRH assay and the HI test. Alternatively, the neutralising antibody response may last longer when compared with the SRH and HI antibody responses.

At time points where SRH antibodies were measurable, PVNT provided a more homogeneous measurement of the antibody response at the group level. Under the conditions of this study, PVNT appeared to be less advantageous when used at the peak of immunity or for correlation with clinical or virological protection, as it could be less discriminatory than the SRH assay. However, further analyses are warranted.

### 4.3. Comparison of PVNT with SRH and HI

The SRH assay has previously been demonstrated to be more reproducible than the HI test when a reference serum is used [11,25]. Several inter-laboratory studies have shown that the HI test lacks reproducibility and consistency because it uses reagents that are difficult to standardize (such as red blood cells). On the other hand, the SRH assay can be adjusted (e.g., by titrating the amount of virus to use in the assay or the incubation period) in order to ensure that a reference serum falls into the expected range [11,25]. The reproducibility of the PVNT has yet to be addressed in the same way. Results obtained in this study demonstrate that the PVNT allowed the sensitive measurement of neutralising antibodies in all immunized ponies at all time points after immunization. The correlation coefficient (*R*^2^) between the PVNT and SRH values was 0.70 (*p* < 0.0001), which is higher than with HI (0.59) and similar to the correlation found in the pilot study using the A/equine/Sussex/1989 strain [9]. This correlation is lower than previously reported for a virus neutralization test using an ELISA to detect the production of virus proteins and SRH for antibodies specific for two strains of the H3N8 subtype of equine influenza virus (Spearman rank correlation coefficients of 0.882 and 0.901) [26]. This result could be linked to differences in the serum set composition between the two studies, with half of the serums taken three weeks after EIV field infection. Post-infection serum samples represented less than 20% of the serum collection used in the current study. However, correlation coefficients can vary between strains. Trombetta et al. demonstrated correlation coefficients of between 0.69 and 0.86 (Pearson’s *R*) between a microneutralization test and SRH for human influenza viruses [27].

Overall, the PVNT efficiently measures neutralising antibodies, which will greatly supplement results obtained with the HI test and SRH assay (HA binding inhibition and complement-binding antibody responses, respectively). However, a guideline on the utility and use of results obtained from different serological assays in the context of vaccine trials or sero-epidemiological studies may be required, and standardization methods would need to take into account the test/assay purpose.

### 4.4. Comparison of Heterologous Strains in PVNT and SRH Assays

The PV used in this study presented the HA only of the A/equine/Richmond/1/2007 strain. In this respect, it is similar to the HI test in only measuring antibodies specific for the HA protein. This would be appropriate for measurement of antibodies to the ProteqFlu vaccine used in group 6, as this expresses only the HA protein of EIV in a canarypox virus background. The SRH assay measures antibodies against the NA also, which will contribute to the reduction of viral load during infection. However, PVs displaying both the HA and NA proteins of EIV have been successfully generated [10].

Vaccination with the heterologous strain (A/equine/Kentucky/2009) used in groups 3 and 4 did not appear to have a significant effect on the measurement of antibodies in the PVNT. It is believed that cross-reactive epitopes in the stalk of the HA may be exposed in PVs because the HA protein is less densely packed on the surface of PVs than in the native virus [28]. In contrast, because the HI test measures antibodies that block the receptor binding site, it is more strain-specific. There was also no significant effect of vaccine strain on SRH antibody levels, which could be explained by that fact that the SRH assay measures a different type of antibody (complement-mediated lysis), and not just those against HA.

## 5. Conclusions

Overall, PVNT antibody responses were in good agreement with SRH/HI antibody responses. Antibodies are measurable by PVNT at time points when the SRH or HI antibody response is of low amplitude, which could greatly improve our understanding of the kinetics of the antibody response to equine influenza vaccination and infection. Further analyses will be required to determine if this observation is linked to technical (e.g., sensitivity) or biological (e.g., antibody response kinetics) reasons. In conclusion, the PVNT efficiently measures neutralising antibodies after immunization or experimental infection in the natural host, which will greatly supplement HA binding inhibition (HI) and complement-binding antibody (SRH) responses. 

## Figures and Tables

**Table 1:** Details of serum set #1. HAU/mL = haemagglutinin (HA) unit per mL.

Group	*n* (Ponies)	*n* (Samples)	Vaccine
1	5	20	A/equine/Richmond/1/2007 (FC2; 400 HAU/mL)
2	5	20	A/equine/Richmond/1/2007 (FC2; 100 HAU/mL)
3	5	20	A/equine/Kentucky/2009 (FC1; 400 HAU/mL)
4	5	20	A/equine/Kentucky/2009 (FC1; 100 HAU/mL)
5	4	16	None (control group)
6	3	12	Commercial vaccine ^1^
**Total:**	**27**	**108**	

^1^ ProteqFlu (Merial) contains A/equine/Ohio/2003 (FC1) and A/equine/Richmond/1/2007 (FC2).

## Supplementary Materials

The following are available online at https://www.mdpi.com/2076-393X/8/3/466/s1, Table S1: Serum set #2 results.
