# Supplementary material for: Evaluation of a Pseudotyped Virus Neutralisation Test for the Measurement of Equine Influenza Virus-Neutralising Antibody Responses Induced by Vaccination and Infection
Source: Vaccines (Basel). 2020 Aug 21;8(3):466. doi: 10.3390/vaccines8030466 (PMC7565038; doi:10.3390/vaccines8030466)
Supplement: Supplementary file 1 [file vaccines-08-00466-s001.pdf]

Evaluation of a pseudotyped virus neutralisation test for measurement of equine influenza A virus neutralizing antibody responses induced by vaccination and infection

Rebecca Kinsley, Stéphane Pronost, Manuelle De Bock, Nigel Temperton, Janet M. Daly, Romain Paillot and Simon Scott

Supplementary Table S1: Serum set #2 results.

**Sample**	**HI titre**	**HI titre (log)**	**SRH titre (mm^2)**	**VN titre (log IC50)**
1	256	2.4	149.0	6.95
2	128	2.1	136.0	5.56
3	512	2.7	171.9	9.15
4	512	2.7	169.6	6.40
5	1028	3.0	181.5	5.91
6	256	2.4	193.9	5.97
7	64	1.8	140.3	5.65
8	256	2.4	144.6	5.94
9	1028	3.0	191.4	5.86
10	256	2.4	123.6	5.86
11	128	2.1	146.8	6.81
12	64	1.8	155.7	5.84
13	128	2.1	198.9	5.58
14	512	2.7	144.6	11.61
15	512	2.7	138.1	5.90
16	64	1.8	149.0	6.17
17	512	2.7	191.4	5.90
18	512	2.7	160.3	5.78
19	64	1.8	100.4	5.79
20	64	1.8	102.2	5.69
25	256	2.4	162.6	5.95
26	1028	3.0	204.1	5.96
21	8	0.9	133.9	4.53
22	16	1.2	109.8	4.18
23	64	1.8	138.1	4.94
24	8	0.9	98.6	3.94
